# Response of hemorrhagic bullous skin lesions of the breast secondary to primary systemic amyloidosis to a five-drug combination chemotherapy: a case report and review of the literature

**DOI:** 10.1186/2162-3619-1-19

**Published:** 2012-08-13

**Authors:** Aref Agheli, Marvin Becker, Gary Becker, M Rashid Chaudhry, Jen C Wang

**Affiliations:** 1 Division of Hematology/Oncology, Brookdale University Hospital & Medical Center, Brooklyn, NY, 11212, USA; 2 Department of Family Practice, Brookdale University Hospital & Medical Center, Brooklyn, NY, 11212, USA; 3 Department of Ear Nose and Throat, Brookdale University Hospital & Medical Center, Brooklyn, NY, 11212, USA

## Abstract

Two major types of amyloidosis are primary amyloidosis or amyloid light chain amyloidosis and secondary amyloidosis. Although amyloidosis involves a variety of organ systems including skin, the occurrence of bullous skin lesions is rare. Little is known about the mechanism of blister formation. These blisters are often hemorrhagic and typically occur in the oral mucosa. Only a few case reports have described skin involvement in systemic amyloidosis. The manifestation of bullous lesions on the breast in association with primary amyloidosis has not been previously reported. Therefore, we report a case of cutaneous hemorrhagic bullous of the breast secondary to primary systemic amyloidosis, which may be important for medical oncologists to be aware of this uncommon presentation of plasma cell dysrasias. Furthermore, this case only partially responded to the commonly used multiple myeloma-type regimen, the skin lesions responded completely to a five-drug combination chemotherapy regimen, utilizing immunomodulators, liposomal doxorubicin, cyclophosphamide, bortezomib, and dexamethasone, suggesting that a more aggressive modality of chemotherapy may be necessary to treat such cases.

## Background

Primary and secondary amyloidosis are the two major types of amyloidosis. The primary systemic amyloidosis, also known as light chain (AL) amyloidosis, is mostly related to a plasma cell dyscrasia. The secondary (AA) amyloidosis is derived from serum amyloid A subunit protein, an acute-phase protein that is produced in response to inflammatory conditions [1]. There is no identifiable, underlying cause of AL amyloidosis [2]. The fibrils of AL amyloidosis are composed of polymerized immunoglobulin light chain or light chain fragments [3]. Amyloid protein is resistant to proteolysis and has a three dimensional configuration as a beta pleated sheet [4]. Although cutaneous involvement in primary systemic amyloidosis is relatively common [5], the occurrence of bullous skin lesions is rare [2,3].

Only a few cases of cutaneous involvement with systemic light chain amyloidosis have been reported in the literature. Skin involvement in the form of hemorrhagic bullous is much rarer. To the best of our knowledge, hemorrhagic bullous presentation of amyloidosis on the breast skin has not been reported in the literature. This uncommon presentation of amyloidosis was only partially responsive to the commonly used combination chemotherapy regimens, but it responded completely to a five-drug combination regimen. This suggests that a more aggressive approach, with combination of multiple immunomodulators and chemotherapy agents may be required to achieve a meaningful response in similar cases.

## Case report

**Figure 1:**
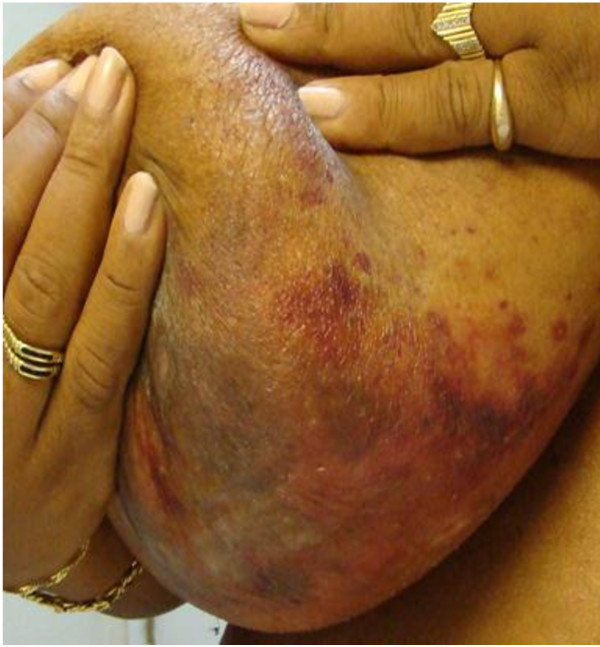
Extensive hemorrhagic bullae with desquamation and surrounding purpura in skin of both breasts, prior to treatment.

**Figure 2:**
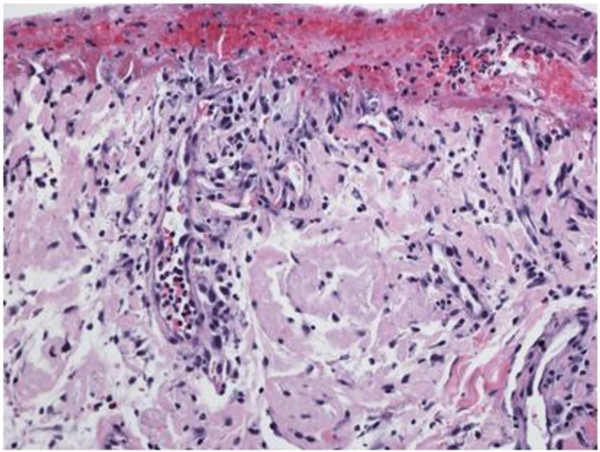
A hematoxylin and eosin stained section demonstrates collections of dull eosinophilic fissured material within the dermis consistent with amyloid, which, on Congo red stain, showed apple-green birefringence in polarized light and confirmed amyloid deposits (not shown).

A 51-year-old African American female with no significant past medical history presented in April 2009 with a 1-month history of hemorrhagic skin lesions on both breasts. The patient had no other symptoms. She was not taking any medication, and her social and family histories were noncontributory. Physical examination revealed extensive bullous ulcerating and hemorrhagic skin lesions involving posterior aspects of both breasts and upper abdominal skin bilaterally (Figure 1). An initial diagnostic skin biopsy of the skin lesion revealed abundant amyloid deposits with positive congo red stain and positive apple-green birefringence under polarized light microscopy. These findings were consistent with pathologic diagnosis of bullous amyloidosis of skin (Figure 2). A direct immunofluorescence study of the specimen with a panel of four immunoglobulins (IgG, IgA, IgM, and C3) was negative. No circulating antibody against basement membrane zone antibody was detected by indirect immunofluorescence study. Initial hematologic workup included serum protein electrophoresis, which had a normal pattern without any M-spike in the gamma region; serum immunofixation was negative for any monoclonal gammopathy, and quantitative immunoglobulin assay was consistent with mild panhypogammaglobulinemia. However, a serum-free light chain assay revealed a very high level of kappa light chain of 6090 mg/dl and lambda light chain of 0.05 mg/dL; urine light chain assay was confirmatory with a very high level of kappa light chain of 6220 mg/dl. The patient also had a mild, normochromic, normocytic anemia with hemoglobin of 11.2 g/dl and normal red blood cell indices. Bone marrow aspiration and biopsy showed infiltration of the marrow with a monoclonal population of plasma cells, comprising 50% of total cells. Flow cytometry of an aspirated bone marrow specimen yielded a monoclonal population of CD138 positive, IgG κ plasma myeloma cells. Conventional cytogenetic examination showed a normal female karyotype of 46 XX; however, FISH was positive for monosomy of chromosome 13 (loss of both RB1 and LAMP1) in 10.3% of cells, and t [6,7], indicating overexpression of BCL1, and cyclin D1 (CCND1/IGH) rearrangement in 5.8% of the cells. Bone survey revealed multiple lytic bone lesions, including the left greater femoral trochanter, right humeral head, and T12 vertebral bodies. Therefore, a diagnosis of bullous hemorrhagic skin lesions, associated with primary systemic light chain amyloidosis was made.

**Figure 3:**
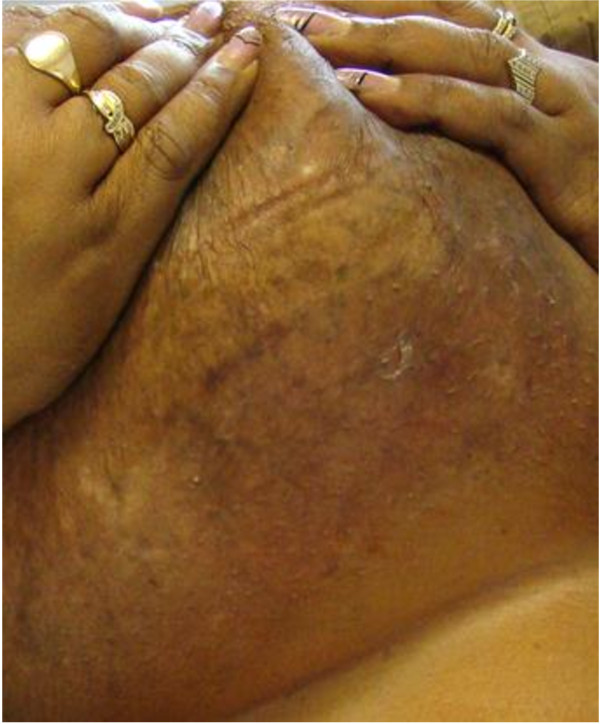
Complete resolution of skin lesions, Post-therapy.

The patient was initially treated with an RVD regimen, which included a combination of bortezomib, with a dose of 1.3 mg/m^2^ on days 1, 4, 8, 11, plus lenalidomide, with dose of 25 mg daily on days 1–14, and dexamethasone, with a dose of 40 mg twice a week, repeated on every 21 days cycles for a total of four cycles. This unfortunately resulted in only a partial response, which included some improvement of the skin lesions and reduction of kappa light chain only to 2250 mg/dl (PR, as per NCCN guideline; more than 50% reduction in the serum free light chain levels). Because of significant persistent skin lesions, a search for enrolling the patient in a clinical trial was attempted; unfortunately, there was none available at the time. Therefore, a more aggressive treatment plan was initiated. This was included a combination of liposomal doxorubicin, with a dose of 40 mg/m^2^ on day one, cyclophosphamide, with a dose of 750 mg/m^2^ on day one, bortezomib, with a dose of 1.3 mg/m^2^, on days 1, 8, and 15, lenalidomide , 15 mg daily on days 1–21 , and dexamethasone, with a dose of 40 mg twice a week, repeated on every 28 days cycles for a total of six cycles. This treatment resulted in a very good partial response rate, with significant reduction of the kappa light chain to 208 mg/dl after two cycles of treatment (VGPR, as per NCCN guidelines; more than 90% reduction in the serum free light chain levels). Continuation of the above regimen for a total of six cycles resulted in further reduction of kappa light chain to 114 mg/dl and complete disappearance of hemorrhagic bullous skin lesions (Figure 3). It is important to mention that although skin rashes are common in the patients treated with lenalidomide, this patient did not report any such adverse side effects with the treatment. The patient was then successfully consolidated with high dose chemotherapy, followed by autologous hematopoietic stem cell transplantation. She has been followed closely, and she has continued to be in complete remission (CR) up to the date of this report.

## Discussion

Bullous pemphigoid (and linear IgA disease) is a heterogeneous group of autoimmune blistering disorders of the subepiderm and is associated with autoantibodies, predominantly IgG, against the transmembrane hemidesmosomal protein BP180/type XVII collagen [8]. Mucous membrane pemphigoid is characterized by subepithelial separation and deposition of immunoglobulins and complement along the basement membrane zone. This disease is diagnosed with direct immunofluorescence testing showing a linear deposition of immunoglobulins and/or complement along the basement membrane zone and indirect immunofluorescence testing showing circulating IgG (and sometimes IgA) autoantibodies along the basement membrane zone [9].

Light chain (AL) amyloidosis is a plasma cell dyscrasia characterized by the pathologic production and deposition of monoclonal light chain fibrillar proteins in tissues, resulting in organ dysfunction. The goal of treatment of AL amyloidosis is eradication of the monoclonal plasma cell population and suppression of the pathologic levels of light chains. The use of novel agents, such as thalidomide, lenalidomide and bortezomib alone or in combination with steroids and alkylating agents has shown efficacy and continues to be explored [10]. HevyLite™ is a new, recently developed method that facilitates separate quantification of the kappa and lambda-bounded amounts of a given immunoglobulin. This new assay provides precise quantification of heavy and light chain and their ratio of the involved immunoglobulin more accurately than the other currently available methods; moreover, they carry prognostic information regarding survival in multiple myeloma patients [11].

Primary systemic amyloidosis, a disease characterized by infiltration of different organs [4], and associated amyloidosis cause clinically detectable cutaneous changes in approximately 10-40% of patients [5]; The occurrence of bullous skin lesions, however, is rare [2,3]. The bullae may be intradermal or subepidermal [2]. The bullae form of cutaneous amyloidosis results from cleavage of the amyloid deposits. These blisters are often hemorrhagic and most commonly occur in the oral mucosa [5].

In this reported case, skin lesions did not reveal immunoglobulin deposits, and indirect immunofluorescence staining for basement membrane protein was negative. Skin biopsy showed positive congo red staining, and amyloid deposits were identified with a positive apple-green birefringence under polarized light microscopy. These findings, along with bone marrow infiltration by abundant plasma cells, multiple lytic bone lesions, and the presence of light chain protein, confirmed a diagnosis of bullous skin lesion secondary to primary systemic amyloidosis.

Insufficient data are available in the literature for the optimal treatment of amyloidosis with underlying plasma cell disorder; therefore, most treatment strategies used in systemic light chain amyloidosis are derived from multiple myeloma regimens. Intermediate or high-dose melphalan followed by stem cell transplantation is one of the therapeutic options listed by the National Comprehensive Cancer Network panel. In some cases, AL amyloidosis is considered to be a treatable disease, and a substantial proportion of patients now attain a long survival; a 22% survival rate of more than 10 years has been reported by Gertz et al. [4]. Treating systemic AL amyloidosis has recently evolved with the use of autologous stem cell transplantation and the introduction of the newer thalidomide analogue. The thalidomide analog, lenalidomide, and the proteasome inhibitor, bortezomib, are both active in advanced and refractory multiple myeloma. The ability of these drugs in combination with dexamethasone to rapidly reduce the level of the monoclonal protein is being examined in several clinical trials. However, this option may not be applicable to all cases, especially those involving hemorrhagic bullous amyloidosis (Table 1). Multiple different single-agent or combination regimens have been used with variable responses, mostly unsatisfactory. Steroids and alkylating agents and even the CHOP regimen (cyclophosphamide, hydroxydaunomycin, Oncovin, and prednisone) have been used as part of the treatment. Lenalidomide is an appealing therapeutic option for subjects with advanced AL amyloidosis and refractory to both melphalan and bortezomib [12].

**Table 1:** Summary of the therapeutic regimens used for the treatment of dermatologic manifestations of systemic AL amyloidosis, by the date of publication

**Author**	**Clinical Syndrome**	**Skin Involvement**	**Reference Published**	**Year**	**Treatment**	**Outcomes**
1. Beachman et al [5]	Diffuse Bullous Amyloidosis of skin	Upper Extremities	(Am Acad Dermatol)	1980	Melphalan + Prednisone	No Clinical CR, 9 mo
2. Ruzicka et al [6]	Bullous amyloidosis	Generalized Skin	(Brit J Dermatol)	1985	Dapson/Prednisone CR	
3. Bieber et al [13]	Hemorrhagic Bullous Amyloidosis	Multiple skin folds	(Arc Dermatol)	1988	Melphalane + Prednisone	Lesions healed with milia
4. Johnson et al [2]	Non-Hemorrhagic Bullous Amyloidosis	Trunk & Proximal Extremities	(CUTIS)	1989	Prednisone + Azathioprine	Treatment failed
5. Pramatarov et al [14]	Multiple Hemorrhagic skin Lesions with systemic Amyloidosis	Multiple skin lesions	(Intern J Dermatol)	1990	Cochicine + DMSO	Partial response
6. Robert et al [3]	Bullous Amyloidosis of skin, 3 cases	Multiple skin	(Medicine)	1993	Alkylating Agents	Poor Response
7. Grundmann et al [7]	Extensive Hemorrhagic Bollous AA-Amyloidosis	Generalized Skin	(Eur J. Med)	2000	Dexamethasone Stabilized after 3 cycles	
8. Ochiai et al [15]	Bullous Pemphigoid Amyloidosis of Hands Systemic AA-amyloidosis	Hands & Feet	(J Cutan Pathol)	2001	CHOP	---
9. Comenzo et al [16]	Review Article Systemic Amyloidosis	---	(Blood)	2002	Melphalane + ASCT	100-day mortality of 21-39 %
10. Gertz et al [17]	Primary Systemic Amyloidosis	Systemic/Skin	(Am J Medicine)	2002	ASCT	Treatment-related Mortality of 14 %
11. Goodman et al [18]	AL Amyloidosis	Systemic/skin	(Brit J Haem)	2006	HD chemotherapy + 100-day mortality ASCT	23 %
12. Giovanni et al [12]	Advanced AL	Systemic/Skin Amyloidosis	(52nd ASH Meeting)	2010	Lenalidomide + Dexamethasone Mortality 13 %	No CR, PHR 41 %,

CR = Complete Response, PHR = Partial Hematologic Response.

It is important to know that 43% of the patients with amyloidosis, who are treated with lenalidomide, may develop skin rashes. The rashes are characterized as morbilliform, urticarial, dermatitic, acneiform, and undefined. Severe rashes require permanent discontinuation of lenalidomide [19].

This unique reported case of AL amyloidosis presented with hemorrhagic bullous skin lesions, high levels of light chain protein only, presence of t(11,14) by cytogenic study, overexpression of cyclin D1, and only partial response to the standard multiple myeloma regimen, which includes lenalidomide and bortezomib, and dexamethasone (RVD). All these features represent a poor prognosis [20-22]. A more aggressive treatment approach with combination of five agents resulted in achieving meaningful success. This included combination of lenalidomide, bortezomib, liposomal doxorubicin, cyclophosphamide, and dexamethasone. The overall response included a very good partial response (VGPR), associated with a great than 90% reduction in the kappa light chain level, and complete resolution of the breast skin lesions. This case illustrates that bullous hemorrhagic skin lesions, associated with primary systemic amyloidosis may need more aggressive treatment such as the five-drug combination that we have used.

## Consent

The patient of the interest in this manuscript has been aware of our intention to publish her case. She has consented to publishing the manuscript without including any pertinent information, which may disclose her identity. We have done our best to protect her identity in this paper.

## Competing interests

No authors report conflicts of interest with the pharmaceutical companies.

## Authors’ contributions

AA, First Author, collected the patient information, reviewed the literature, and drafted the manuscript, and revised the final manuscript. JCW, Corresponding Author, designed the treatment protocol, and attended the patient; assisted and mentored in the writing the manuscript. MB, assisted in treating the patient, and assisted in collecting the relevant clinical data. GB, assisted in treating the patient, and assisted in collecting the relevant clinical data. GRC, assisted in treating the patient, and assisted in collecting the relevant clinical data. All authors have given approval for the final approval of the version to be published.
